# Screening for *Mycobacterium tuberculosis* Infection Using Beijing/K Strain-Specific Peptides in a School Outbreak Cohort

**DOI:** 10.3389/fcimb.2021.599386

**Published:** 2021-03-18

**Authors:** Ji Young Hong, Ahreum Kim, So Yeong Park, Sang-Nae Cho, Hazel M. Dockrell, Yun-Gyoung Hur

**Affiliations:** ^1^Department of Pulmonary and Critical Care Medicine, Hallym University Medical Center, Gangwondo, South Korea; ^2^Institute for Immunology and Immunological Diseases, Yonsei University College of Medicine, Seoul, South Korea; ^3^Department of Infection Biology, London School of Hygiene and Tropical Medicine, London, United Kingdom

**Keywords:** Beijing/K strain, cytokine, IFN-γ release assay, latent tuberculosis infection, *Mycobacterium tuberculosis*, outbreak

## Abstract

**Background:**

The Beijing strain of *Mycobacterium tuberculosis* (*M. tb*) has been most frequently isolated from TB patients in South Korea, and the hyper-virulent Beijing/K genotype is associated with TB outbreaks. To examine the diagnostic potential of Beijing/K-specific peptides, we performed IFN-γ release assays (IGRA) using a MTBK antigen tube containing Beijing/K MTBK_24800, ESAT-6, and CFP-10 peptides in a cohort studied during a school TB outbreak.

**Methods:**

A total of 758 contacts were investigated for *M. tb* infection, and 43 contacts with latent TB infection (LTBI) and 25 active TB patients were enrolled based on serial screening with QuantiFERON-TB Gold In-Tube tests followed by clinical examinations. Blood collected in MTBK antigen tubes was utilized for IGRA and multiplex cytokine bead arrays. Immune responses were retested in 24 patients after TB treatment, and disease progression was investigated in subjects with LTBI.

**Results:**

Total proportions of active disease and LTBI during the outbreak were 3.7% (28/758) and 9.2% (70/758), respectively. All clinical isolates had a Beijing/K *M. tb* genotype. IFN-γ responses to the MTBK antigen identified *M. tb* infection and distinguished between active disease and LTBI. After anti-TB treatment, IFN-γ responses to the MTBK antigen were significantly reduced, and strong TNF-α responses at diagnosis were dramatically decreased.

**Conclusions:**

MTBK antigen-specific IFN-γ has diagnostic potential for differentiating *M. tb* infection from healthy controls, and between active TB and LTBI as well. In addition, TNF-α is a promising marker for monitoring therapeutic responses. These data provide informative readouts for TB diagnostics and vaccine studies in regions where the Beijing/K strain is endemic.

## Introduction

The Beijing family of *Mycobacterium tuberculosis* (*M. tb*) is a predominant genotype in East Asia, with hyper-virulence and rapid transmission (Glynn et al., 2002). It has been reported as an important risk factor for treatment failure or relapse of tuberculosis (TB) (Burman et al., 2009). The Beijing strains have caused large outbreaks of TB and extensive spread of multi-drug resistant TB in several countries (Glynn et al., 2002; Gurjav et al., 2016). South Korea is an intermediate TB burden country, with 66 cases per 100,000 population reported in 2018 (WHO, 2018). About 70% of clinical isolates in South Korea belong to the Beijing family, and Beijing/K was identified as a predominant Beijing genotype that causes pulmonary TB outbreaks (Kim et al., 2001). The Beijing/K strain of *M. tb* rapidly replicates and demonstrates severe pathogenic features at an early stage of infection in mice compared with *M. tb* H37Rv (Kim et al., 2017). Whole genome sequencing of the Beijing/K strain revealed a 6-kDa early secretory antigenic target (ESAT-6)-like protein, MTBK_24800 (GenBank accession no. AIB49024.1), within the 5.7-kb insertion region compared with the H37Rv (Han et al., 2015; Hur et al., 2016). More than 95% identical amino acid sequences were found in other clinical isolates, such as GuangZ0019 and CDC1551, which are super-extensively drug resistant and have been highly transmissible during outbreaks in China and USA (Valway et al., 1998; Lin et al., 2013; Hur et al., 2016). However, there is no sequence similarity to genes in *M. bovis* Bacillus Calmette–Guérin (BCG; Pasteur 1173P2, Denmark 1331, Moreau RDJ) and nontuberculous mycobacteria (NTM), such as *M. avium* and *M. marinum* (Hur et al., 2016). Diagnostic values of IFN-γ responses induced by recombinant MTBK_24800 protein were demonstrated by differentiating *M. tb*-infected groups from healthy controls (Hur et al., 2016). Several epitope sites of MTBK_24800 were predicted and the predominant epitope site was found at C-terminal amino acids (76-99) in active TB patients (Hur et al., 2016).

A third of the population in South Korea has latent TB infection (LTBI) although the prevalence of LTBI has gradually decreased: 59.3%, 44.4%, and 33.2% in 1975, 1990, and 2016, respectively (Cho, 2018). Remarkable decrease of LTBI prevalence was reported in the younger population: 6.5% and 10.9% in the ages 10 to 19 and 20 to 29, respectively (Yeon et al., 2018). Based on recent surveillance data using Interferon (IFN)-γ release assays (IGRA), the estimated prevalence among first grade high school students (mean age 15.3) was 2.1% (Yeon et al., 2018). The IGRA such as QuantiFERON-TB Gold In-Tube (QFT-GIT) test is used to screen TB contacts for *M. tb* infection in the field. This commercial IGRA using ESAT-6 and 10 kDa-culture filtrate protein (CFP-10) is advantageous compared to the conventional tuberculin skin test because it has no cross-reactivity with *M. bovis* BCG vaccination or infection with most NTM (Pai et al., 2014). However, its variable outcome in the borderline range of cut-off points (Metcalfe et al., 2013; Jonsson et al., 2017) and insufficient differentiation between active TB and LTBI (Hur et al., 2016) suggest the need for an improved antigen-specific biomarker.

Here, we report the investigation of a recent TB outbreak that occurred from September to November 2017 among students at a high school in South Korea. Case detection and contact tracing were performed by clinical evaluation, chest radiography, and QFT-GIT test. Based on epidemiologic investigation, contacts with active disease and LTBI were enrolled to examine IFN-γ responses induced by *M. tb* using Beijing/K-specific MTBK_24800 and ESAT6/CFP-10 peptides. The potential diagnostic values of 6 selected peptides, two from each antigen, were assessed compared to the commercial QFT-GIT peptide pool, and the antigen-specific IFN-γ responses were followed up in patients with TB after treatment. IFN-α, interleukin (IL)-13, IL-17, C-X-C motif chemokine 10 (CXCL10), C-C Motif Chemokine Ligand 5 (CCL5), and tumor necrosis factor (TNF)-α were also measured.

## Materials and Methods

### Epidemiologic Investigation and Enrolment of Study Subjects

**Figure 1:**
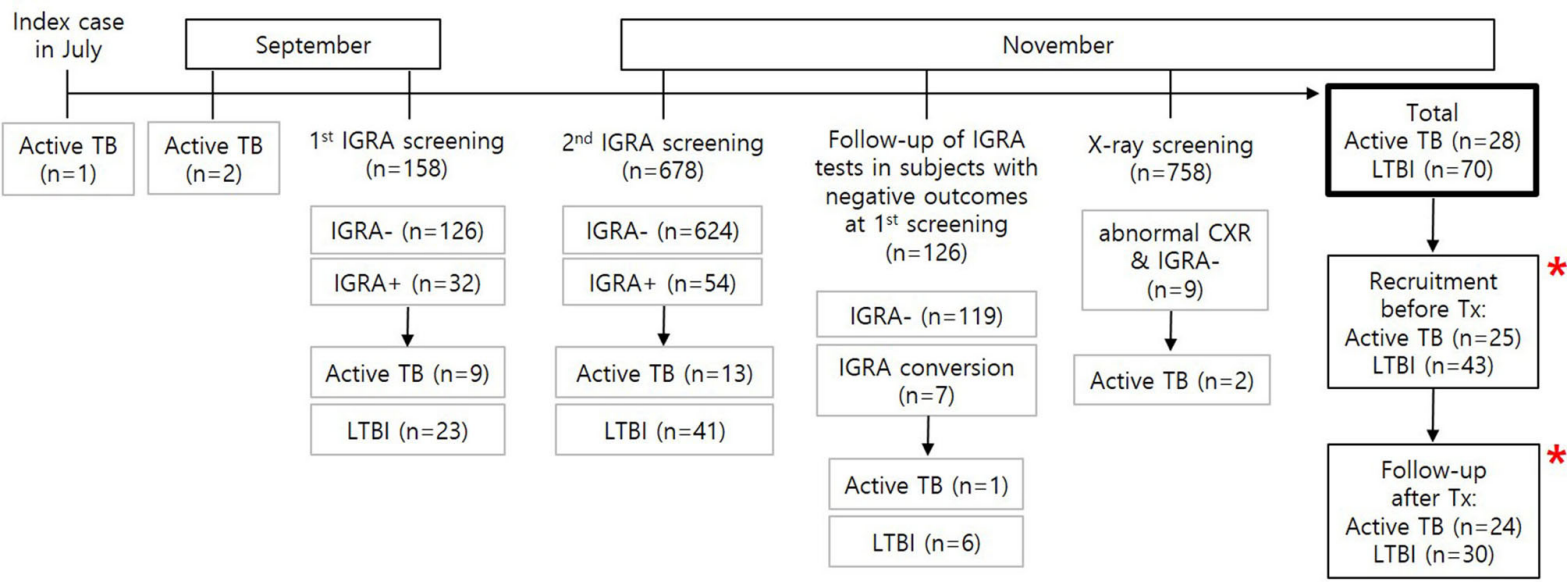
Flowchart of *M. tb* infection screening during the TB outbreak and recruitment of study subjects. A total of 28 active patients with TB and 70 individuals with LTBI were found based on contact screening tests for *M. tb* infection in 758 individuals. Among the contacts, 25 TB patients and 43 subjects with LTBI were recruited for this study. Immune responses were followed up in 24 patients with TB and 30 subjects with LTBI after successful treatment. * MTBK antigen tubes were used.

The first index case developed a productive cough and fever in July 2017. He had been treated for Mycoplasma pneumonia but was finally diagnosed as having sputum smear positive-pulmonary TB in September 2017. As two additional active TB cases were identified in the same high school, TB contact surveillance was conducted through an epidemiologic investigation (**Figure 1**). Diagnosis of active TB cases was confirmed by chest x-rays, sputum-smears using auramine fluorochrome staining, and culture using solid (3% Ogawa) and liquid (BACTEC MGIT 960, Becton Dickinson, Sparks, MD, USA) media. Nucleic acid probes (Gen-Probe, San Diego, CA, USA) were used to identify *M. tb* complex from samples that showed mycobacterial growth.

The TB contact investigation used QFT-GIT and chest X-rays. High-resolution chest CT scans were performed in subjects with positive IGRA results to help differentiate active TB from LTBI. Initial screening by QFT-GIT in 158 close contacts who were at high risk of infection diagnosed 9 active TB and 23 LTBI cases. Following further testing of a total of 678 students and staff at the school in November 2017, 13 contacts were diagnosed with active TB and 41 with LTBI. Considering that QFT-GIT has a window of conversion after exposure to *M. tb* (Anibarro et al., 2011), a second QFT-GIT test was performed after 8 weeks in 126 close contacts with initially negative IGRA results. Seven subjects had IGRA conversion, and one was confirmed to have active TB. Two of 9 subjects with abnormal chest X-ray and negative IGRA results were diagnosed with active TB; the remaining six subjects were confirmed to have no abnormal parenchymal lesion by chest CT, and one subject was diagnosed with bacterial pneumonia and cured after antibiotic treatment. In total, 28 patients with active TB and 70 individuals with LTBI were diagnosed (**Figure 1**).

The TB patients were treated using the first line standard regimen using rifampicin, isoniazid, ethambutol, and pyrazinamide for 6-9 months. The LTBI contacts received prophylactic therapy with rifampicin and isoniazid for 3 months. Based on the results of screening for *M. tb* infection, 70 contacts with LTBI were advised to take prophylactic treatment. Among 69 subjects who started treatment, 59 successfully completed 3 months of treatment, and 10 discontinued treatment due to elevated liver enzymes or change of mind. One student who refused LTBI prophylaxis treatment progressed to active TB after 3 months and received anti-TB medication for 6 months. Of 43 LTBI subjects who participated in this study, 7 discontinued prophylactic treatment. However, none has progressed to active disease in the following 2 years.

Prior to anti-TB treatment, 25 active TB patients and 43 LTBI contacts provided written informed consent for inclusion in the present study, in accordance with the Declaration of Helsinki (**Figure 1**). Consent of legal guardians was obtained if participants were younger than 19 years. Subjects who agreed to the follow-up study were enrolled after treatment, comprising 24 active TB and 30 LTBI subjects. Students who showed negative IGRA results were not allowed to visit the hospital, and 24 normal healthy controls who had no history of previous *M. tb* exposure were recruited outside the school, as negative controls. Since the healthy control group was not recruited from TB outbreak cohort, age and gender distributions were not similar to that of active TB and LTBI groups. Healthy controls were older than patients with active TB and LTBI subjects (median age: 38 in health controls, 18 in active TB, 18 in LTBI). More females were included in the healthy control group, compared with the active TB and LTBI subjects (healthy controls: 83.3%, active TB:0%, LTBI: 2.3%). None of the study participants had HIV, cancer or diabetes. The study was approved by the Institutional Review Boards of Chuncheon Sacred Heart Hospital (approval number: 2017-27).

### Preparation of MTBK Antigen Tube Including Beijing/K-Derived Peptides

Peptide antigens (without glycosylation) were designed based on predicted epitope sites for major histocompatibility complex type II binding; the predicted binding affinity was scored according to different types of human leukocyte antigen, and the epitope sites with highest scores were chosen from the database of SYFPEITHI program (Rammensee et al., 1999). Peptides were synthesized with > 95% purity (GenScript, NJ, USA) and each peptide stock (1mg/mL) was dissolved in distilled water, following in RPMI1640 media for working solution. The MTBK antigen tube was formulated with peptides containing two dominant epitope sites, each from the *M. tb* ESAT-6 (45 µg/mL), CFP-10 (45 µg/mL), and MTBK_24800 (10 µg/mL) proteins (Rammensee et al., 1999; Arend et al., 2000; Aagaard et al., 2009; Hur et al., 2016) (**Table 1**). Nil tubes including RPMI 1640 media only were also prepared.

**Table 1:** Amino acid sequences of *M. tb* antigens.

Antigen	Amino acid	Sequence
ESAT-6	1-20	MTEQQWNFAGIEAAASAIQG
	59-83	DATATELNNALQNLARTISEAGQAM
CFP-10	1-25	MAEMKTDAATLAQEAGNFERISGDL
	48-69	GTAAQAAVVRFQEAANKQKQEL
MTBK_24800	30-47	EARRMWASSQNISGAGWS
	76-99	RDGLVRDANNYEQQEQASQQILSS

Predicted dominant epitopes were selected from M. tb ESAT-6, CFP-10, and MTBK_24800, and six different synthetic peptides were combined in a tube (MTBK).

### Preparation of Blood Samples

Heparinized blood samples were collected in three QFT-GIT tubes (QuantiFERON-TB Gold; Qiagen, Hilden, Germany) and in MTBK tubes containing ESAT-6, CFP-10, and MTBK_24800 peptides. The QFT-GIT and MTBK tubes with blood samples were incubated upright at 37°C for 18-24 hours, and the supernatants were harvested. All samples were stored at -70°C until the end of subject recruitment.

### QFT-GIT IFN-γ Enzyme-Linked Immunosorbent Assays (ELISA)

Harvested supernatants were utilized for IFN-γ ELISA according to the manufacturer’s protocol (QuantiFERON-TB Gold; Qiagen, Hilden, Germany). Briefly, 50 µL of freshly prepared conjugate solution, reconstituted standards, and harvested plasma samples were loaded into 96-well microplates. The microplates were incubated at room temperature for two hours, and 100 µL of enzyme substrate solution was added to each well after washing the microplates six times using diluted wash buffer. Finally, 50 µL of enzyme stopping solution was added with a 30-minute incubation at room temperature, and the microplates were measured at 450 nm to obtain optical density readings.

### Multiplex Bead Array

Six analytes (IFN-α, IL-13, IL-17A, CXCL10, CCL5, TNF-α) were simultaneously measured using a multiplex bead array kit according to the manufacturer’s protocol (Merck Millipore, Darmstadt, Germany). Assay plates were measured on a Luminex100 machine (Luminex, Austin, TX, USA), and a standard curve for each analyte was obtained using MasterPlex QT 2010 software (Hitachi, San Bruno, CA, USA).

### Molecular Genotyping of Clinical Isolates

*M. tb* isolates were obtained from 6 of 10 TB patients who showed *M. tb* growth in culture and Beijing/K strain type confirmed by polymerase chain reaction (PCR) (Warren et al., 2004). Briefly, genomic DNA was extracted from each isolate, and PCR was performed using Prime Taq polymerase (Genetbio Inc., Daejeon, Korea) and primer sets to identify 4 particular genotypes: Beijing (5’-GACIIICCGGGGCGGTTCA-3’, 5’-CATGAACTCGCGGCTGTTTAGG-3’), Beijing/K (5’- GACIIICCGGGGCGGTTCA-3’, 5’-GGAGCGATGAACGACTAGAGCAC-3’), Beijing/M (5’-CGTCGTGCCCGTATAACCG-3’, 5’- GACIIICCGGGGCGGTTCA-3’), and Non-Beijing (5’-GTGGTGAGACGCGTAGTTCG-3’, 5’-GTACGGCGAAAGGTTTGAGC-3’). Sets were designed to determine positive amplification of ~350bp, ~250bp, ~150bp, and ~500bp, respectively. PCR reaction mixtures were denatured at 95°C for 5 minutes, annealed at 62°C for 20 seconds, and extended at 72°C for 30 seconds for 35 cycles. Negative control did not include genomic DNA.

### Statistical Analysis

An optimal cut-off point for positive IFN-γ responses was determined based on Youden’s index greater than 70% for both sensitivity and specificity (Fluss et al., 2005). Diagnostic accuracy and significance of immune responses were analyzed by the area under the ROC curve (AUC), one-way ANOVA with subsequent Kruskal-Wallis test, and Wilcoxon signed rank test. Agreement of the results between antigen tubes (MTBK vs. QFT-GIT) was measured using Kappa statistics.

## Results

### Characteristics of Study Participants From the School Outbreak Cohort

The general characteristics of participants are described in **Table 2**. Age and sex were similarly distributed between TB patients and LTBI contacts. All participants had received childhood BCG vaccination and had no other acute or chronic disease. Ten (40%) of the TB patients were positive by respiratory specimen culture, and 15 were diagnosed by radiologic and clinical criteria. Most patients were diagnosed early after exposure, and few had cavities or more than two-thirds chest field extension. All the TB patients were treated with first-line anti-TB drugs for more than 6 months, and one with tuberculous pleurisy underwent surgical pleural decortication. All the TB patients showed radiologic and clinical improvement with negative culture results at completion of therapy.

**Table 2:** Characteristics of study subjects from the school outbreak cohort.

	Active TB (n=25)	Latent TB (n=43)
Age†	18 (17-18)	18 (17-18)
Male	25 (100%)	42 (97.7%)
BMI (kg/m^2^)^†^	21.8 (19.7-24.2)	22.8 (20.6-25.4)
BCG vaccination	25 (100%)	43 (100%)
IGRA positivity	23 (92%)	43
Smear AFB positivity	1 (4%)	
*M. tuberculosis* culture positivity	10 (40%)	
Extrapulmonary TB	1 (4%)	
Chest image at diagnosis		
Cavity	2 (8%)	
Centriolobular nodules	23 (92%)	
Pleural effusion	1 (4%)	
Extension of chest field		
Less than one-third	23 (92%)	
Less than two-thirds	0 (0%)	
More than two-thirds	2 (8%)	

The number of subjects for each feature is noted. All but one patient had pulmonary TB, and only the patient with extrapulmonary TB showed AFB positive result at diagnosis. Ten of 25 (40%) TB patients were confirmed to have M. tuberculosis growth by culture, whereas most subjects (92%) showed positive IGRA results.

^†^Median (interquartile range IQR).

BMI, body mass index; AF, acid fast bacilli.

### *M. tb* Genotypes of Clinical Isolates

**Figure 2:**
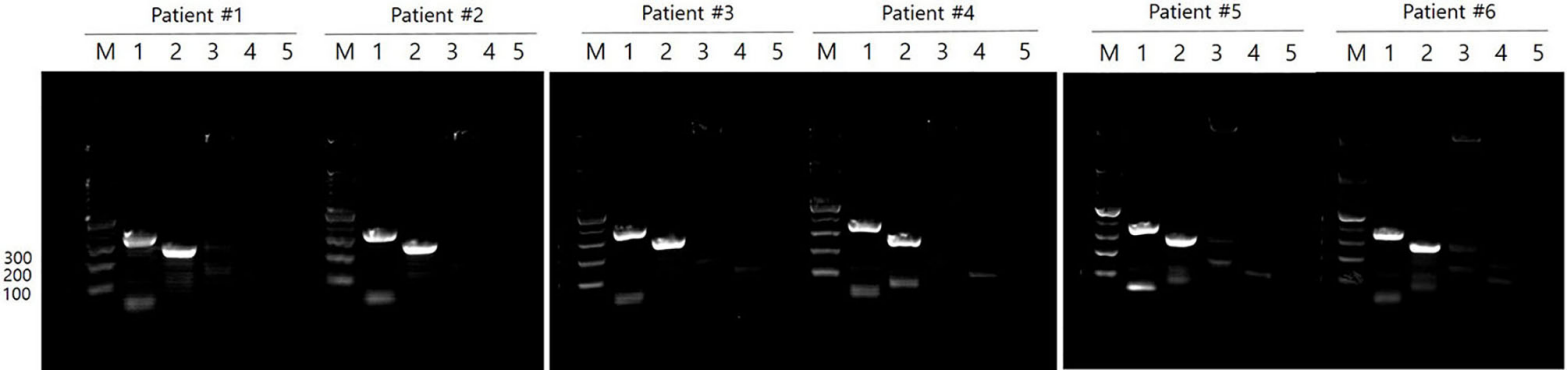
*M. tb* genotypes in clinical isolates. *M. tb* genotypes were confirmed by PCR in 6 of 10 patients who showed *M. tb* growth in culture. All clinical isolates were Beijing/K strains of *M. tb*. M: 100 bp DNA ladder, Lane 1: Beijing strain (~ 350 bp), Lane 2: K strain (~ 250 bp), Lane 3: M strain (~ 150 bp), Lane 4: Non-Beijing strain (~ 500 bp), Lane 5: negative control.

Clinical isolates were obtained from 6 of 10 patients for whom *M. tb* growth was confirmed in culture. Based on PCR analysis, the *M. tb* Beijing (350 bp) and K (250 bp) genotypes were demonstrated in all clinical isolates tested (**Figure 2**).

### Change of QFT-GIT Test Outcomes at Diagnosis and Enrollment

*M. tb* infection was diagnosed by QFT-GIT IGRA screening during the contact investigation, and those with positive IFN-γ results were subsequently examined for diagnosis of active TB in the hospital at which the study subjects had been enrolled. Study participants including active TB patients were retested for *M. tb* infection by QFT-GIT during recruitment, and the two test outcomes were analyzed. Reversion of IFN-γ results was observed in four contacts in the LTBI group during the 3-5 weeks between the initial IGRA screenings and retesting at recruitment (**Supplementary Figure 1**). In contrast, only one patient showed IGRA conversion. All the IFN-γ values with outcome differences were less than 0.7 IU/mL, except for one LTBI contact who had a value of 5.03 IU/mL IFN-γ at diagnosis (**Supplementary Figure 1**).

### IFN-γ Responses to MTBK Antigen

**Figure 3:**
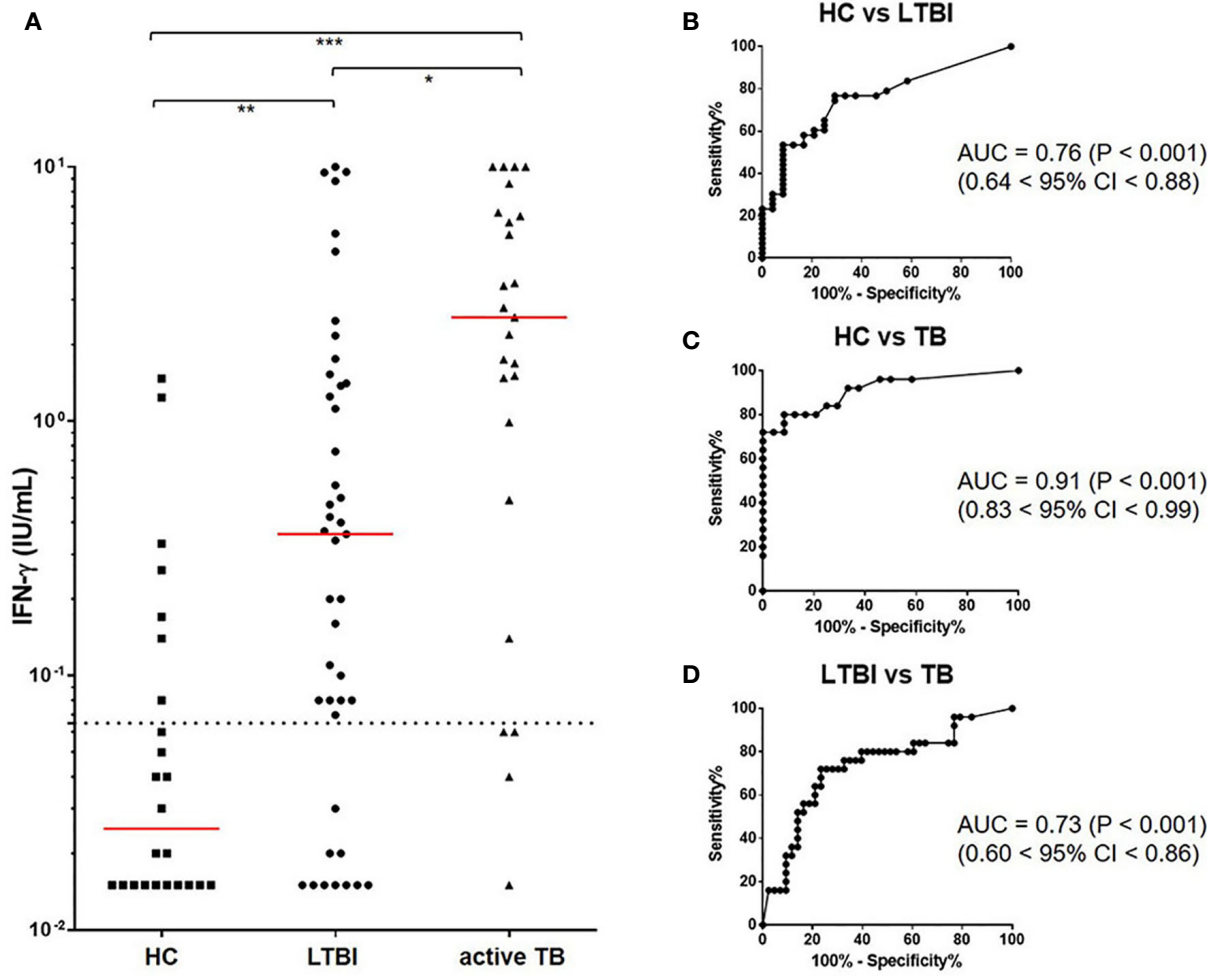
IFN-γ responses to the Beijing/K-peptide combination and diagnostic accuracy of identifying *M. tb* infection. IFN-γ responses to MTBK antigen were significantly higher in active TB and LTBI compared with HC. *M. tb* infection and disease status were differentiated by IFN-γ responses. The median value of IFN-γ is marked in red, and a dotted line represents the cut-off for positive results (0.065 IU/mL) **(A)**. Immune responses to the MTBK antigen had good diagnostic values (AUC > 0.7) for distinguishing between active TB and LTBI **(D)**, as well as between infected and non-infected individuals **(B, C)**. *P < 0.05, **P < 0 .01, ***P < 0.001 by one-way ANOVA with subsequent Kruskal-Wallis test, HC, healthy control.

MTBK antigen comprised of 6 peptides, including two dominant epitopes from each of MTBK_24800, ESAT-6, and CFP-10 (**Table 1**). IFN-γ responses to the MTBK antigen differentiated between non-infected control and both infected groups, LTBI and active TB (P<0.01 and P<0.001, respectively) (**Figure 3A**). The optimal cut-off value for IFN-γ positivity was 0.065 IU/ml IFN-γ, which had 79% sensitivity (68% < 95% CI < 88%) and 71% specificity (49% < 95% CI < 87%). Based on this positive cut-off point (> 0.065 IU/ml), approximately 77% (33 of 43) of the contacts showed positive IFN-γ responses, and the positive rate was slightly higher in TB patients than in contacts with LTBI (83%; 19 of 23 patients). The AUC value for detecting LTBI was 0.76 (0.64 < 95% CI < 0.88, P<0.001), but the diagnostic accuracy was increased in TB patients (AUC = 0.91, 0.83 < 95% CI < 0.99, P<0.001) (**Figures 3B, C**, respectively). The MTBK antigen-specific IFN-γ responses also differentiated between active TB and LTBI (AUC = 0.73, 0.60 < 95% CI < 0.86, P<0.001) (**Figures 3A, D**).

### Comparative Analyses of IFN-γ Responses Between the Two Sets of Antigen Tubes

Based on the rates of IFN-γ positivity and negativity, total concordance of the results between the MTBK antigen tube and the QFT-GIT antigen tubes was about 81% (kappa=0.23, positive agreement: 89%, negative agreement: 32%). Among 68 enrolled subjects, 13 (3 patients and 10 individuals with LTBI) showed different results to the two antigen tubes. Two of 5 subjects with negative IFN-γ responses by QFT-GIT showed positive results in the IFN-γ ELISA using the MTBK antigen.

### Multiple Cytokine Responses to Beijing/K-Peptide Combination

**Figure 4:**
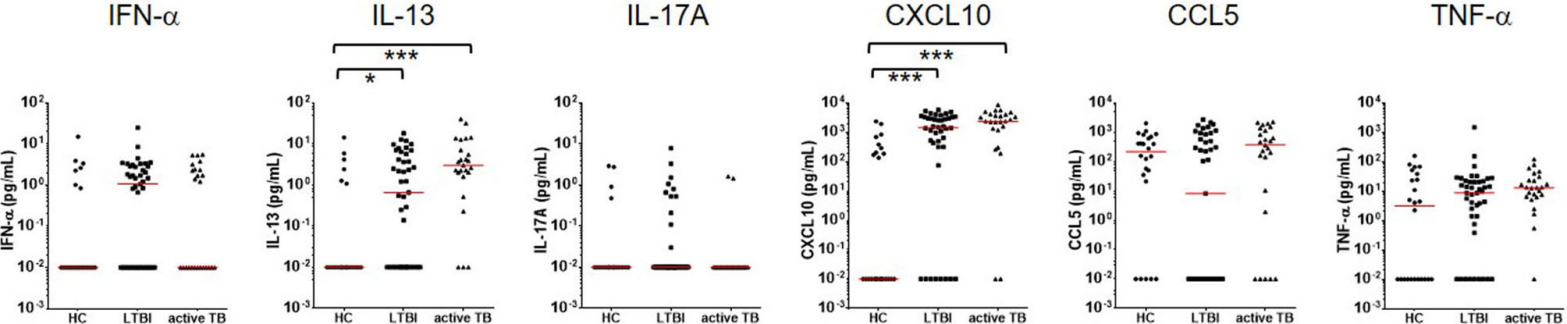
Measurement of multiple cytokine responses to Beijing/K-combination. Among IFN-α, IL-13, IL17, CXCL10, CCL5, and TNF-α, the IL-13 and CXCL10 responses were significantly higher in LTBI and active TB compared with HC (antigen minus nil). However, disease status (active TB vs. LTBI) was not differentiated by the cytokine/chemokine responses. The median responses are marked by red lines. *P < 0.05, ***P < 0.001 by one-way ANOVA with subsequent Kruskal-Wallis test, HC, healthy control.

To test whether cytokines other than IFN-γ might provide improved diagnostic accuracy, additional six cytokines and chemokines were assayed in the culture supernatants. None of the cytokine and chemokines tested (IFN-α, IL-13, IL-17, CXCL10, CCL5, and TNF-α) differentiated between active TB and LTBI, unlike IFN-γ in response to the MTBK antigen. However, IL-13 and CXCL10 responses were significantly higher in infected contacts than healthy controls (**Figure 4**). Diagnostic accuracy was better at identifying active TB than LTBI for both IL-13 and CXCL10 responses (AUC: 0.83 vs. 0.69 for IL-13, 0.90 vs. 0.79 for CXCL10, P<0.001). IFN-α, IL-17A, CCL5 and TNF-α responses to the MTBK antigen did not vary between the healthy controls and *M. tb* infected groups (**Figure 4**).

### Immune Responses With or Without Antigen Stimulation Following TB Treatment

**Figure 5:**
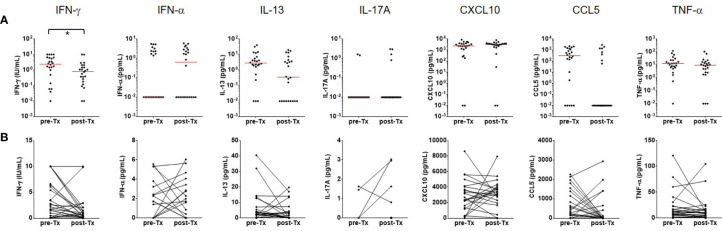
Follow-up immune responses to the Beijing/K-peptide combination after TB treatment. **(A)** Distribution of cytokines and chemokines as immune responses to the Beijing/K-peptide combination after TB treatment. The median responses are marked by red lines. **(B)** Longitudinal analysis of cytokine and chemokines after TB treatment. Significant decreases in IFN-g response to the MTBK antigen were observed in 24 TB patients who finished anti-TB treatment. Other cytokines and chemokines tested in this study did not show significant changes after treatment (antigen minus nil). *P < 0.05 by Wilcoxon signed rank test, Tx, treatment.

**Figure 6:**
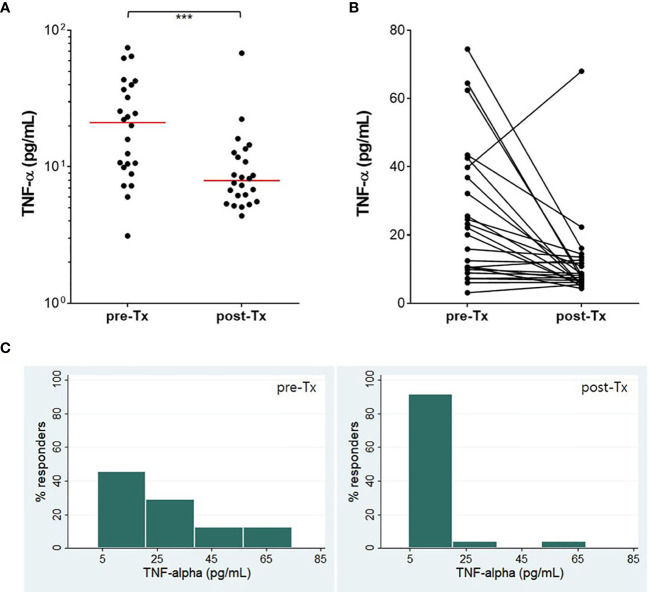
Treatment efficacy measured by TNF-α. TNF-α concentration (nil) was significantly reduced after TB treatment **(A, B)**. The proportion of responders with a high level of TNF-α decreased, while that of responders with a low level of TNF-α dramatically increased **(C)**. The horizontal red line represents the median value of TNF-α. ***P < 0.001 by Wilcoxon signed rank test, Tx, treatment.

The IFN-γ, IFN-α, IL-13, IL-17, CXCL10, CCL5, and TNF-α responses were followed in 24 patients who finished TB treatment. A significant decrease (P<0.05) of the IFN-γ response was found after TB treatment although IFN-γ values did not revert to negative in most patients (**Figure 5**). The proportion of strong IFN-γ (≥ 1.5 IU/mL) responders was reduced, and about 75% of responders had low IFN-γ responses (≤ 1.0 IU/mL) after TB treatment. The other six analytes did not show significant changes in response to the antigenic stimulation. However, spontaneous TNF-α (P<0.01) release was significantly reduced after TB treatment (**Figure 6**). The proportion of responders with spontaneous high levels of TNF-α release was also decreased with therapy, whereas more patients had weak positive TNF-α responses (**Figure 6**).

## Discussion

TB is one of the top 10 causes of death and the leading cause from a single infectious agent (WHO, 2019). The End-TB strategy of WHO to end the global TB epidemic focuses on early detection, treatment, prevention, and intensified research including development of effective vaccines (WHO, 2015). In South Korea, BCG vaccination is strongly recommended after birth, but the value of the BCG vaccine is limited by insufficient protection against pulmonary TB and by variable efficacy against different *M. tb* strains reported in diverse geographic locations (Kaufmann et al., 2015). Therefore, there would be clinical value in developing an intervention strategy or new diagnostic tool by focusing on the highly virulent *M. tb* strains found in regional TB outbreaks. In this study, all the tested clinical isolates had a Beijing/K genotype, supporting the previous finding that the Beijing/K genotype of *M. tb* is most frequently associated with TB outbreaks in South Korea (Kim et al., 2001). IFN-γ responses to the MTBK antigen were significantly higher in TB contacts than healthy controls, and also differentiated between active TB and LTBI. Diagnostic accuracy was demonstrated by an AUC value (> 0.7) that distinguished *M. tb* infection from healthy controls and defined disease status (active TB vs. LTBI). Anti-TB treatment resulted in reduced MTBK antigen-specific IFN-γ responses and decreased plasma TNF-α production. These results suggest that measurement of IFN-γ induced by the MTBK peptides may have promise as a TB diagnostic assay and may improve the current IGRA based on ESAT-6 and CFP-10.

Delayed identification of the index case seemed to cause the primary case in September, resulting in rapid transmission of infection with prolonged contact within the school. AFB staining of sputa is generally utilized but does not distinguish between *M. tb* and NTMs (TDIUATLD, 2005). *M. tb* growth can be confirmed by culture, but it takes at least 4 weeks to achieve results unless a faster molecular assay such as GeneXpert is used (TDIUATLD, 2005). In this study, only one patient showed a positive AFB smear result at the early diagnosis time point, and culture positivity was later identified in about 40% of patients. However, 92% (23 of 25) of the TB patients showed positive IFN-γ responses at the time of initial screening, indicating that the blood test may help in early diagnosis of TB in those with negative AFB results.

The QFT-GIT is a very important recent advance in TB diagnostics although it does not have sufficient value to differentiate between active TB and LTBI or to predict treatment efficacy and disease cure (Clifford et al., 2015). Moreover, it is not robust enough to diagnose *M. tb* infection at borderline cut-off ranges (Metcalfe et al., 2013; Jonsson et al., 2017). IGRA conversion and reversion were also found in this study, in which two individuals whose IGRA reverted had values close to the cut-off point, supporting that the QFT-GIT test outcome is not confirmatory near the cut-off point. This suggests the need for a new generation of IGRAs with additional or alternative antigens and cytokines/chemokines. A recent prospective study demonstrated greater than 80% sensitivity and specificity for detecting active TB using IGRAs with the *M. tb* Rv3615c and Rv3879c antigens (Whitworth et al., 2019). An ESAT-6-free IGRA antigen cocktail had good diagnostic performance in a high TB burden area (Nemes et al., 2019), indicating that it might be considered as an assay for diagnosis in trials with future ESAT-6-containing vaccines. Our data showed that MTBK_24800 peptides with ESAT-6 and CFP-10 which are main antigens of QFT-GIT differentiated between active TB and LTBI.

IFN-γ is strongly associated with protective immunity against TB, but many other cytokines and chemokines are also crucial for control of *M. tb* infection. In the early response to *M. tb*, TNF-α synergizes with IFN-γ to activate macrophages and eliminate ingested bacteria (Zuñiga et al., 2012). With increased production of Th2 cytokines, IL-13 inhibits autophagy-dependent killing of *M. tb* (Deretic et al., 2009), whereas pro-inflammatory IL-17 participates in granuloma formation by recruiting activated neutrophils (Okamoto et al., 2010). CXCL10 has good diagnostic value for detecting active TB in children and adults, but it does not distinguish between active TB and LTBI similar to IFN-γ measured by QFT-GIT (Whittaker et al., 2008). Recently, CXCL10 has been suggested as a biomarker for monitoring pulmonary TB along with CCL5 (Zhao et al., 2018). In this study, significantly higher IL-13 and CXCL10 responses were found in *M. tb*-infected groups compared with healthy controls whereas only IFN-γ differentiated between active TB and LTBI. The IFN-γ responses were also significantly decreased with reduced TNF-α concentrations in cured TB patients. These findings suggest that IFN-γ is the most robust biomarker for identifying *M. tb* infection in response to the Beijing/K peptide combination, whereas the spontaneously released plasma TNF-α may predict treatment efficacy or disease cure. Based on the report that IFN-α production is related to the virulence of an *M. tb* strain (Manca et al., 2001), it is expected that the IFN-α responses might be significantly different in ESAT-6/CFP-10 free cocktails containing only peptides from Beijing/K-specific antigens.

This study has some shortcomings, that could be addressed in future TB outbreaks. Firstly, not all the TB patients were positive by the gold-standard *M. tb* culture, and as a result *M. tb* isolates were not available from all the tested TB patients to confirm that they were infected with the same Beijing strain of *M. tb*. Secondly, due to limited blood samples, the new MTBK_24800 peptides were not tested on their own, but only in combination with peptides from ESAT-6 and CFP-10. The improved diagnostic potential of this peptide mix can now be dissected further using MTBK_24800 peptides on their own in comparison with the selected ESAT-6 and CFP-10 peptides. Although the MTBK antigen differentiated between active TB and LTBI, there is no evidence which the MTBK antigen improved the diagnostic performance of QFT-GIT test in this study. For the comparison, two types of antigen tubes should be prepared (MTBK_24800 with the antigens of QFT-GIT vs. QFT-GIT alone).

The relatively low cut-off point (0.065 IU/ml, > 70% sensitivity and specificity) for MTBK-specific IFN-γ and the overlapping values between the healthy control and LTBI groups should be examined further to meet the required standard for clinical use. In particular, although an unrelated group of adult healthy controls were used, not from the school where the outbreak occurred, additional testing in healthy Korean controls is needed. A newer generation of QuantiFERON test (QFT-Plus) contains ESAT-6, CFP-10 and several peptides to stimulate both CD4- and CD8 T cells. The sensitivity of QFT-Plus in active TB patients was 1.3% higher, compared with QFT-GIT, suggesting that more epitope peptides in QFT-Plus antigen may get higher IFN-γ levels (Oh et al., 2020). Thus upgraded antigen cocktails including additional epitopes from MTBK_24800, optimization of the peptide concentrations, and ESAT-6/CFP-10 free peptide cocktails should be prepared and tested in further prospective cohort studies with a large number of patients. However, to the best of our knowledge, this is the first study to report screening for *M. tb* infection using an IGRA with an antigen derived from a predominant *M. tb* outbreak strain (Beijing/K) in a TB outbreak cohort. The immune signature of the IGRA may therefore provide useful information for the development of improved TB diagnostics and vaccines where the Beijing/K strain of *M. tb* is endemic.

## Data Availability Statement

The raw data supporting the conclusions of this article will be made available by the authors, without undue reservation.

## Ethics Statement

The studies involving human participants were reviewed and approved by the Institutional Review Boards of Chuncheon Sacred Heart Hospital (approval number: 2017-27). Written informed consent to participate in this study was provided by the participants’ legal guardian/next of kin.

## Author Contributions

Y-GH and S-NC designed the conception of the research. JH enrolled study participants and followed up TB patients after treatment. JH, SP, AK, and Y-GH performed the preparation of samples and experiments. Y-GH analyzed and interpreted the data. Y-GH and JH wrote the original draft. HD reviewed the draft and served as a scientific advisor. All authors contributed to the article and approved the submitted version.

## Funding

This study was supported by the National Research Foundation of Korea grant (NRF-2018R1D1A1A02049260) funded by the Ministry of Education, and the Bio & Medical Technology Development Program (NRF-2017M3A9E8033225) in South Korea.

## Conflict of Interest

The authors declare that the research was conducted in the absence of any commercial or financial relationships that could be construed as a potential conflict of interest.
