# Supplementary material for: Screening for Mycobacterium tuberculosis Infection Using Beijing/K Strain-Specific Peptides in a School Outbreak Cohort
Source: Front Cell Infect Microbiol. 2021 Mar 18;11:599386. doi: 10.3389/fcimb.2021.599386 (PMC8044942; doi:10.3389/fcimb.2021.599386)
Supplement: Supplementary file 1 [file Image_1.pdf]

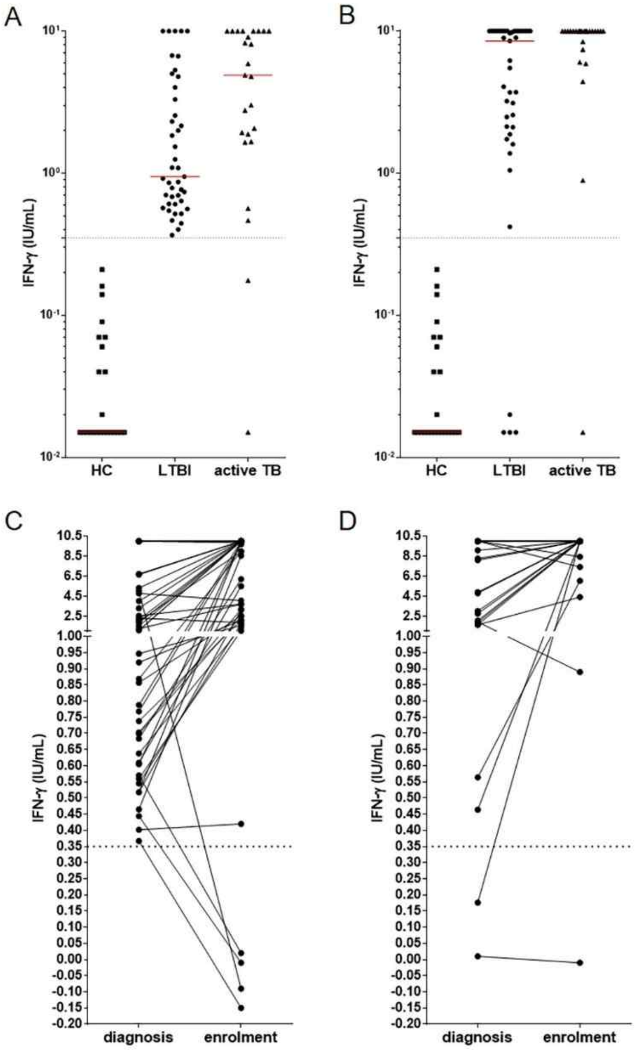

Supplementary Figure 1. **Changes of QFT-GIT test outcomes.** The first QFT-GIT test was performed at diagnosis as part of the epidemiological screening (A) and study subjects were retested at enrolment (B). IFN-γ positivity by QFT-GIT test reverted to negative in four subjects with LTBI at the 2nd screening (enrolment point) (C). In contrast, one patient with TB showed QFT-GIT conversion (D). The horizontal red line represents the median value of IFN-γ. The cut-off for positive results (0.35 IU/mL) is marked by a dotted line.
